# Incorporating a Ferrous Polymer Target into Elastomeric Liners for Socket Fit Sensing in Prosthesis Users

**DOI:** 10.3390/s20195620

**Published:** 2020-10-01

**Authors:** Ryan V. Carter, Brian G. Larsen, Jake B. McLean, Joseph L. Garbini, Joan E. Sanders

**Affiliations:** Departments of Bioengineering and Mechanical Engineering, University of Washington, 3720 15th Ave NE, Box 355061, Seattle, WA 98195-5061, USA; rvcarter@uw.edu (R.V.C.); bglars@uw.edu (B.G.L.); jakebrooksmc@gmail.com (J.B.M.); garbini@uw.edu (J.L.G.)

**Keywords:** prosthetics, trans-tibial, amputee, residual limb, inductive sensor, socket fit, distance sensing, interface mechanics, volume management

## Abstract

Liner-to-socket distance measurement using inductive sensing may be an effective means to continuously monitor socket fit in people using trans-tibial prostheses. A practical limitation, however, is a means to incorporate a thin uniform-thickness layer of conductive or magnetically permeable target material into the wide range of prosthetic liner products that people with limb amputation commonly use. In this paper, a method is presented whereby a 0.50-mm thickness ferrous polymer made from a SEEPS polymer and iron powder that is formed adjacent to a 0.25-mm thick non-ferrous layer of SEEPS polymer is assembled between two sheets of elastic fabric material. Bench testing showed that the fabrication procedure achieved a root-mean-square error in the thickness of this construct of 58 μm, helping to create a consistent calibration result over the entire surface. The original fabric backing of an off-the-shelf prosthetic liner was removed and replaced with the developed construct. When worn in the shoe of an able-bodied participant for 7.5 h per day for 28 days, the sensor well maintained the shape of its calibration curve at the start of wear, but a distance offset (shifting of the *y*-intercept) was introduced that increased during the initial approximately 12 days of wear. When the distance offset was corrected, for the primary distance range of clinical interest for this application (0.00–5.00 mm), the sensor maintained its calibration within 4.4%. Before being used in clinical application for liner-to-socket distance monitoring, new ferrous liners may need to be pre-worn so as to achieve a consistent distance reference.

## 1. Introduction

Portable sensors are commonly used in manufacturing applications such as robotics [1,2], but service applications have received an enormous amount of attention in recent years, especially in clinical medical research. Continuous measurement of prosthetic socket fit may improve the quality of life of people who use prosthetic limbs. Communicating socket fit status to users can help them sense how a deterioration in fit feels and train them when to make a socket adjustment (e.g., add or remove socks). Field-collected data can be used as a diagnostic tool for insensate patients to help practitioners identify when and how a socket fit problem started [3]. Another application of continuous socket fit sensing is towards the design of auto-adjusting sockets, sockets that change their size automatically to well maintain socket fit based on sensor data [4].

Efforts to sense socket fit have focused on measurement of limb–socket interface pressure or distance. Distribution of load and movement between the limb and socket would be expected to well reflect how well the prosthesis couples to the residual limb. Innovative technologies using pressure sensing in feedback control systems to auto-adjust socket size have been discussed, some of which have advanced to clinical testing [5,6,7,8,9,10,11]. Optical and inductive sensing methods have been used to detect distance. Optical sensing accurately detects limb–socket distance [12], but the requirement of a line of sight limits practical application. Inductive sensing eliminates the need for a line of sight. Inductive sensing elements have been incorporated into the socket, and a target attached to the user’s prosthetic liner [13,14,15]. An auto-adjusting socket that used an inductive sensor for feedback control maintained socket distance and appropriately responded to a change in set point during testing on amputee participants [4].

Though inductive sensing is a promising technology for continuous measurement of socket fit and implementation in automatically adjusting sockets, practical issues limit its widespread use. While the active element (an antenna that serves as both an oscillator and a detector) is incorporated into the socket material, a relatively straightforward task since sockets are custom fabricated for prosthesis users anyway, the target is not. The target must be incorporated into the wearer’s prosthetic liner, typically an off-the-shelf product. There are numerous liner sizes and styles; most are made of silicone, polyurethane, a thermoplastic elastomer, or a combination of these [16,17]. Most liners have a fabric backing on the outside to allow the liner to easily slip into a socket. Affixing a textile target made of a conductive fabric to the outside of commercial liners was ineffective because of durability issues; the normal and shear stresses experienced at the liner–socket interface during ambulation damaged the fabric and broke the conductive elements [18]. We worked with one prosthetic liner manufacturer to successfully incorporate a material with high magnetic permeability (iron powder) into their liner fabrication process for a single liner model [19]. A magnetically permeable target (e.g., iron powder) is structurally different than a conductive element. While the magnetically permeable design produced excellent results [15], numerous liners are used by the amputee population thus the model selected served only a limited number of users. A necessary next step is to extend from this success to create a more general fabrication technique so that many commercial liners can be made into “ferrous liner” products, increasing the number of prosthesis users who can use a socket with fit sensing. Potentially, insight gained from the present study could be used by manufacturers to incorporate ferrous targets into their product as part of the fabrication procedure. Advancement of fabrication methods should enhance participant eligibility in research studies and speed up the regular use of the technology in clinical practice.

We recently developed a Styrene-Ethylene-Ethylene/Propylene-Styrene (SEEPS) thermoplastic elastomer combined with iron powder (85% wt) that serves as an excellent magnetically permeable target for inductive sensing in lower-limb prostheses [20]. While the ferrous polymer performs well in bench testing, it must be incorporated into users’ liners to be of clinical utility. This is a challenging effort because the ferrous polymer layer as well as the layers external to it (between the target and socket wall) must be of very consistent thickness to ensure consistent performance over the entire liner surface and thus ensure calibration results are not dependent on the rotation about the limb axis of the liner in the socket. In this research we created a method to modify commercial liners into liners with an embedded ferrous target. The techniques for fabrication are described, supplemented by photographs in the Appendix A showing each step of the fabrication process. Results from bench tests are discussed, including repeated testing conducted on samples worn in an able-bodied person’s shoe for 28 days. Data collected from an instrumented socket of a prosthesis user at different sock thicknesses are also presented.

## 2. Materials and Methods

**Figure 1:**
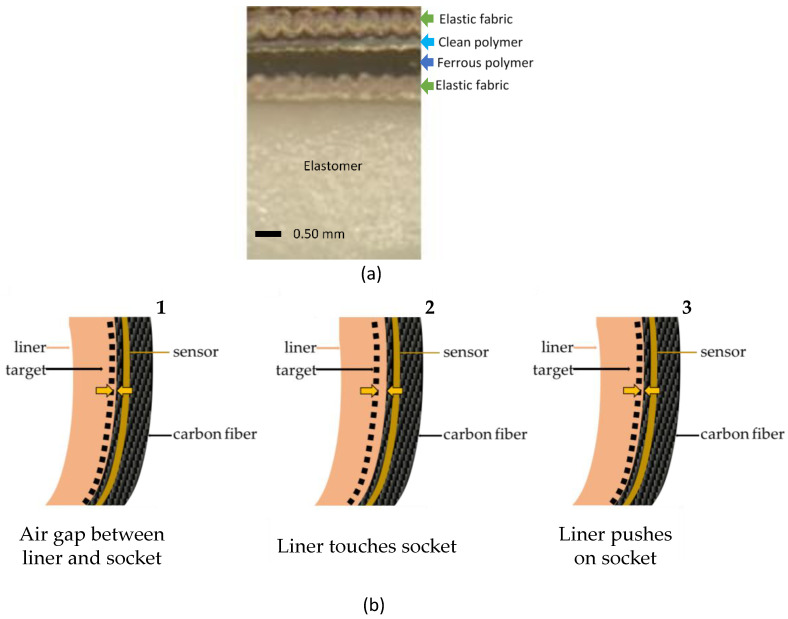
Ferrous liner. (**a**) Cross-section of a ferrous liner immediately after fabrication. (**b**) Three possible configurations of measurement. Orange arrows indicate distance between the target and socket.

We created a technique to integrate a magnetic target into elastomeric liner products available in the prosthetics industry. The fabric backing of the original liner is removed and replaced with a flexible construct that includes a ferrous polymer and an outer clean polymer (Figure 1a). The clean polymer serves as an elastic covering, and the ferrous polymer serves as a target for an inductive sensing element that is placed within the wall of the socket during fabrication (Figure 1b). The inductive sensing element is a custom antenna made using flexible circuit board printing techniques and is embedded approximately 0.6 mm from the inside surface [15].

An inductive sensing chip (LDC1614, Texas Instruments, Dallas, TX, USA) housed in an instrumentation box outside of the socket drives the antenna to make an inductor-capacitor (LC) tank oscillator. The magnetically permeable target (iron) in the liner reinforces the inductor and alters the sensor’s oscillation frequency in a distance-dependent manner. The change in sensor frequency measured by the inductive sensing chip provides a sensitive measure of the distance between the antenna and target [19,20]. This distance is calibrated to the target-to-socket distance, using a custom testing jig described below. As a final step in calibration to correct for the depth of each individual antenna into the socket wall, an inflatable bladder at 27.6 kPa is positioned within the socket and sensor data are collected.

There are three possible configurations of the liner and socket (Figure 1b): (1) the liner and socket do not touch, i.e. there is an air gap between them; (2) the liner is flush with the socket wall; and (3) the residual limb compresses the liner against the socket and the outer elastic layers thin. The sensor detects target-to-socket distance for all three of these configurations. Target-to-socket distance is used as the distance of interest rather than liner-to-socket distance because the clean polymer and elastic fabric are elastic and thus thin under an applied pressure. Calibration to target-to-socket distance avoids negative distance measurements when the liner is compressed.

There are two stages to fabrication of the liner: preparation of the ferrous polymer and clean polymer construct; and attachment of it to the off-the-shelf liner. A detailed illustration of the step-by-step process is provided in Appendix A.

### 2.1. Preparation of the Ferrous Polymer and Clean Polymer Construct

The content of the ferrous polymer layer is identical to that developed in prior research—a SEEPS polymer mixed with iron powder at 85 wt % [20]. An 85 wt % was found previously to produce an acceptable calibration result (sensor counts/mm) for this application while still maintaining tensile and compressive mechanical properties matched to those of commercial elastomeric liners. A tensile property match at the interface of the ferrous polymer and elastomer of the liner helps lower stress concentrations there and reduces the risk of delamination. A sheet of the ferrous polymer (thickness 0.50 mm) is created as described below using a custom mold and commercially available laboratory equipment. The necessary equipment is listed in Table A1 in Appendix A. The substances used to make the ferrous polymer and clean polymer construct are listed in Table 1.

To begin, the base of the mold, which is of inner dimension 38 cm × 53 cm and made of aluminum (6061-T6), is prepared by coating it with a thin layer of mold release agent, covering it with an aluminum lid, and heating it on hot plates set to 285 °C.

**Figure 2:**
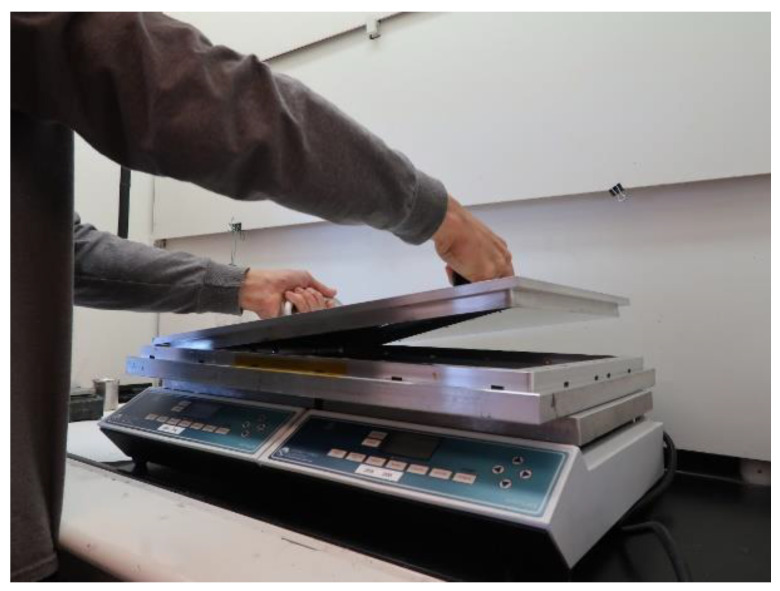
Mold on hotplates. Top lid being positioned in place.

To create the polymer, 10 g of SEEPS copolymer is mixed with 50 g of mineral oil in an insulated cup. The mixture is heated on a hot plate at 265 °C until it is sufficiently melted that it will mix with the iron powder. A total of 340 g of iron powder is added, 30 g at a time, while continually stirring. Once the thick liquid is well mixed, it is degassed for 4 min in a vacuum oven pre-heated to 250 °C at a vacuum pressure of 91.4 kPa. The iron mixture is poured in one smooth motion onto the pre-heated base of the mold coated with the release agent. The lid of the mold is placed on top (Figure 2) and clamped at the edge on the four sides to ensure a uniform pressure. An additional C-clamp is placed at each corner. Once clamped, the frame is removed from the hot plates and cooled for approximately 2 h.

**Figure 3:**
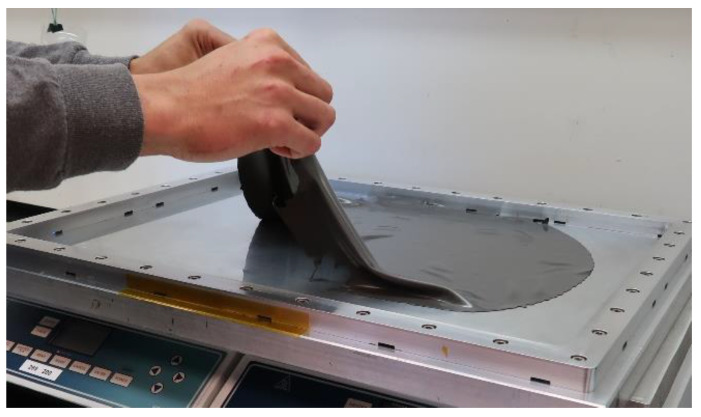
FPPC construct being removed from the mold.

Next, the lid is removed, and the ferrous polymer is left attached to its bottom surface. To create the clean polymer, a 0.25-mm thick layer of SEEPS polymer (no iron), the mold is pre-heated to 200 °C. Ten spacers of height 0.25 mm are placed around the inside rim of the mold. A mass of 15 g of pellets (4044) is mixed with 75 g of mineral oil in an insulated cup and heated on a hot plate to 265 °C. The polymer is degassed in a 250 °C pre-heated oven for 4 min at a vacuum pressure of 91.4 kPa. The polymer is quickly poured onto the base of the mold and spread. The lid with the ferrous layer still adhered to it is placed into the base. The lid is quickly clamped at the edge on the four sides using the same eight clamp positions described above. The mold is removed from the hot plates (Figure 3) and cooled. This assembly is termed the ferrous polymer/polymer construct (FPPC).

### 2.2. Attachment to off-the-Shelf Liner

The fabric backing of many liner products is attached to the elastomer with glue. It can be removed by mechanically peeling off the material, starting at the proximal edge. Both the fabric backing and the glue layers are removed in a single peeling procedure. We have found that excess glue adhering to the elastomer can be removed with a dermatome. The distal umbrella for liners with locking pin suspension is removed and retained.

To cut the FPPC to the proper dimension for the liner to which it is to be attached, the socket worn with the ferrous liner is measured. The distance from the center of the distal end of the socket to 3.5 cm beyond the proximal brim on the posterior aspect is recorded. The FPPC height is cut to this vertical dimension.

A layer of elastic fabric is added to each side of the FPPC before attachment to the liner. The inner layer of elastic fabric provides a means for affixing the FPPC to the elastomer material, which is typically made of silicone, polyurethane, or a thermoplastic elastomer. Without the elastic material, the two surfaces may not bond together well, and delamination may occur during wear. The outer layer of elastic fabric serves to replace the fabric backing material. The FPPC with elastic material on both sides is sandwiched between two large aluminum plates with spacers placed on the inside edge. The spacers are equal to the thickness of the FPPC. The plates are clamped on the four sides using 16 alligator clips. The assembly is placed on hot plates at 200 °C, and then a 111.2 N weight is placed on top for 8 min. The weight is removed, and two large 69 N spring clamps are placed on opposite long sides to hold the assembly together while it is inverted. After it is inverted, the assembly is placed back on the hot plates for 45 s with the 111.2 N weight placed on top.

After the assembly cools the construct is removed, and the elastic fabric that overlaps the FPPC is removed. The sides of the construct are sewn together around the bottom and long side to create the liner shape. The FPPC with the elastic fabric on both sides is termed the elastic FPPC (e-FPPC).

**Figure 4:**
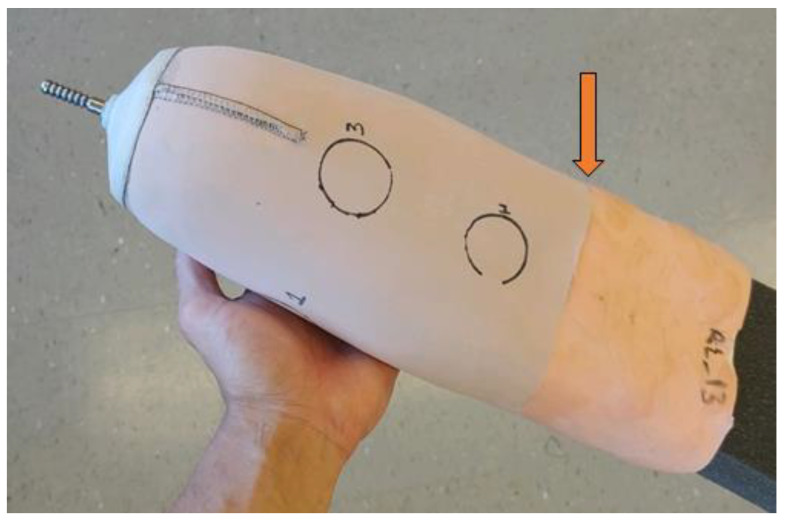
Completed elastomeric liner with an embedded ferrous polymer target. The arrow indicates the proximal edge of the e-FPPC. The circles indicate locations of sensor antennae when the liner is within the user’s socket.

To attach the e-FPPC to the off-the-shelf liner that has had its fabric backing and distal umbrella removed, a silicone adhesive is used. After it is cleaned with isopropyl alcohol and allowed to dry, the liner elastomer is placed over a foam positive (distal end upward) of diameter slightly larger than the liner. Lines of silicone adhesive are applied at a 5.0 cm spacing and run the length from the distal end to the proximal edge. The adhesive is spread into an even layer and then the e-FPPC is inverted and rolled over the outside of the liner. To work the adhesive into a smooth layer, traditional methods used to smooth a prosthetic socket layup are used (e.g., a string around the outside pushed inward and downward to drive the adhesive into a smooth layer). If the liner has locking pin suspension, then a hole is cut through the e-FPPC to allow the locking pin to pass through. The umbrella is re-affixed to the distal end with silicone adhesive. A weight is hung over the distal end to apply compression while the adhesive cures. A complete ferrous liner is shown in Figure 4. The proximal edge of the e-FPPC near the brim of the socket is visible.

### 2.3. Testing

**Figure 5:**
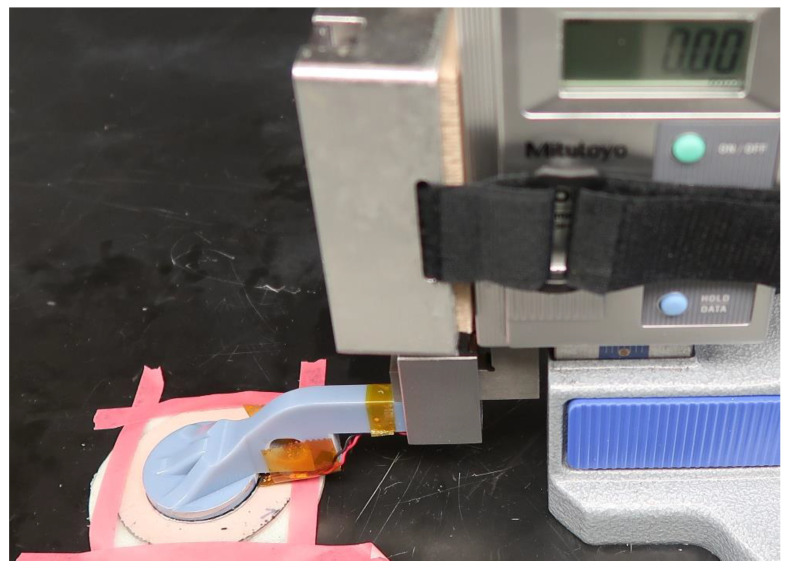
Calibration unit.

Calibration tests were performed using a test setup similar to that described previously [20] (Figure 5). A sensor antenna was mounted to a plastic arm extending laterally from a vertical height gauge (570-312, Mitutoyo, Aurora, IL, USA) (resolution 0.01 mm) such that the sensor was positioned above the e-FPPC. Sensor data and height gauge readings were recorded at increments of 0.25 mm for a sensor distance above the target from 0.00 to 2.00 mm, and at increments of 1.00 mm for distances of 2.00–15.00 mm. Data were also collected with no target present. The value with no target was subtracted from all data within a trial. Samples were tested from five locations on a 38 cm × 53 cm e-FPPC sheet that reflected anterior and posterior regions of interest. To calculate measurement inconsistency across the sheet, we fit calibration data from all five locations with one equation. The equation was the sixth-order polynomial with the smallest least squares error to all of the collected data and was termed the mean calibration. The absolute value of the distance offset of the data at each of the five locations relative to the mean calibration (root mean square error) was calculated. This distance offset corresponded to the deviation in measurement of thickness relative to the mean.

Durability was evaluated by placing one 5.08-cm diameter sample of a ferrous liner (an e-FPPC affixed to a liner elastomer) in the heel of each shoe (traditional running shoes) of an able-bodied participant (77 kg) for 28 days. No extra insoles were worn. The samples were worn during the day while the participant worked in an indoor setting, walking frequently. Steps were recorded using a step counter (ActiLife ActiGraph GT3X+). Calibration tests were conducted at Days 0 (before wear), 1, 3, 7, 10, 14, 18, 21, 24, and 28. Two back-to-back trials were conducted on each of the two samples (one in each shoe).

Clinical testing was conducted on an amputee participant with trans-tibial amputation. Internal Review Board approval was obtained, and a written informed consent form was signed before any test procedures were initiated. The participant walked on a treadmill at a comfortable walking speed wearing an instrumented socket and a ferrous liner. Sensor antennae were embedded within the socket at six locations: anterior proximal near the socket brim; mid-limb at anterior, posterior lateral, and posterior medial locations; and inferior at anterior and posterior locations. The participant conducted six bouts of walking on a treadmill at a self-selected walking speed wearing the ferrous liner and instrumented socket. Each walking bout was approximately 5 min in duration and was preceded by a stand under equal weight-bearing of approximately 5 min. Different liner and sock configurations were worn in different bouts. During Bouts 1, 4, and 5, the participant wore the ferrous liner with no socks. During Bouts 2 and 6, the participant wore the ferrous liner with a three-ply sock. The sock was positioned between the ferrous liner and the socket. During Bout 3, the participant wore a different liner with no socks. The total duration of the testing protocol was approximately 75 min. Sensor signals were converted to distance using calibration data and plotted as a function of time. Shapiro–Wilks tests conducted to evaluate normality showed that data from one of the 5-min walking bouts was not normally distributed, thus Wilcoxon signed rank tests were used for all statistical comparison.

## 3. Results

Results from the five test locations in the 38 cm × 53 cm sheet demonstrated a consistent thickness. The deviation in thickness calculated as the RMS error with respect to the mean was 58 μm.

The samples tested for durability by placing them in the heel of the shoe of an able-bodied participant were worn a mean of 7.5 h (SD 1.4) per day for the 28 days of wear. The samples were subject to a mean of 4049 (SD 1980) steps of walking per day. The total elapsed time between the first day and last day of wear was 53 days. For both samples, the data collected during calibration testing on Day 28 at 0.00 mm height did not follow the calibration curve, suggesting a calibration execution error at 0.00 mm height. Those two points were not included in analysis.

**Figure 6:**
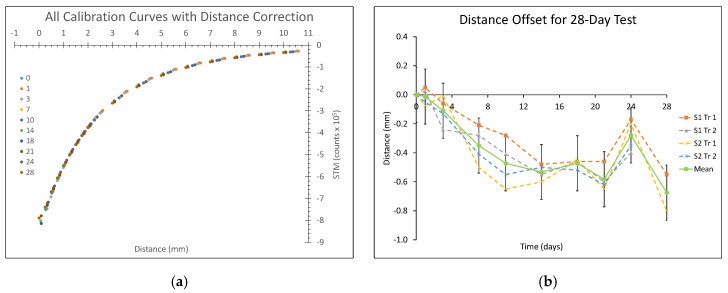
Results from the 28-day durability test. (**a**) Data from all test days corrected for distance offset (left shoe sample (Trial 1)). Results were comparable for both samples. (**b**) Distance offset over time for the first and second trials for both samples and the mean. S1 = left shoe sample; S2 = right shoe sample; Tr1 = Trial 1; Tr2 = Trial 2.

Calibration results (counts/mm) changed over time. From before wear to the last day of wear (Day 28), counts/mm increased between 9.0% and 42.4% depending on the target-to-socket distance. This change was a result of the calibration curve shifting over time to the left. For the 0.00–5.00 mm distance range, the range of primary clinical interest in this application, the change in counts/mm caused an absolute value percent error between the post-Day 0 data and the Day 0 calibration curve of between 0.8% and 33.3% (median 16.4%). The shifting of the calibration curve to the left may have been due to compaction of the outer elastic fabric and polymer layers of the e-FPPC over time. When the data were corrected for this distance offset after Day 0 (i.e., shifting the *y*-intercept of the post-Day 0 calibration curves), it well-aligned for all days (Figure 6a). When the distance offset correction was made, the absolute value percent error between the post-Day 0 data and the Day 0 calibration curve for the 0.00–5.00 mm distance range was between 0.4% and 4.4% (median 2.0%). We further found that the distance offset change decreased approximately linearly over time for about 12 days, and subsequently was more stable (except for one outliner point at Day 24) (Figure 6b).

The participant with trans-tibial limb amputation tested in the lab was 45 years of age and had his amputation as a result trauma seven years prior. His residual limb was of length 12.5 cm from the patellar tendon to distal end of the tibia, and of mid-limb circumference 32.7 cm. His residual limb was fleshy, muscular, and cylindrical in shape. He did not have diabetes or heart disease but did have high blood pressure and took medication for it. The test prosthesis was an endoskeletal carbon fiber patellar bearing socket with direct attachment of a high-performance, carbon fiber blade prosthetic foot. Locking pin suspension was used.

**Figure 7:**
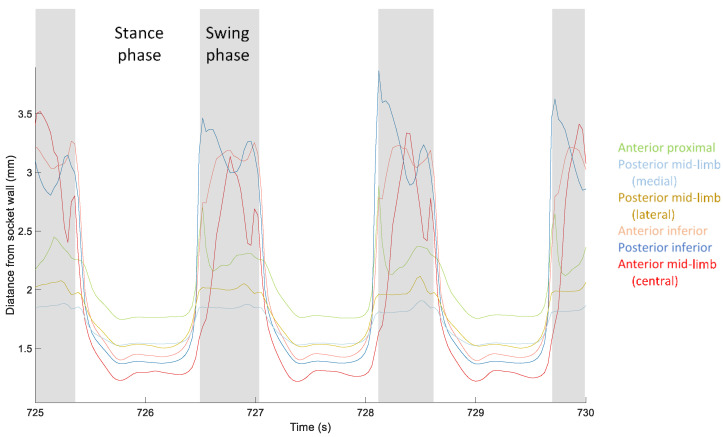
Exemplary data from a participant with trans-tibial amputation. Data from three steps are shown. Stance phase is unshaded, and swing phase is shaded gray.

**Figure 8:**
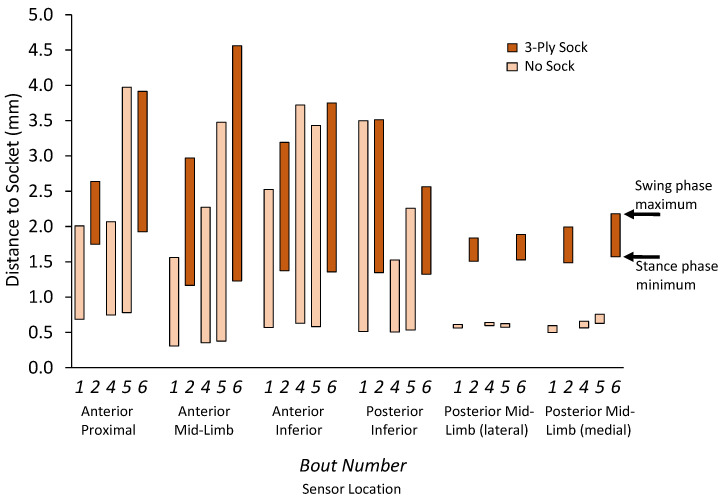
Range of limb–socket distance within a step: swing phase maximum and stance phase minimum. Results from steps wearing a three-ply sock and no sock during a trial are shown.

Target-to-socket distance measurements during stance phase demonstrated a double peak-minima curve, the first peak reflecting weight acceptance and the second peak reflecting push off (Figure 7), consistent with ground reaction force data from traditional gait analysis [21]. Data from steps where the participant wore the three-ply sock demonstrated greater stance phase minima and stance phase maxima than data from steps where the participant did not wear a sock (Figure 8). The mean difference in stance phase minimum (closest distance to the socket wall) between the three-ply sock and no-sock configuration was 0.91 mm (SD 0.10). The difference between the swing phase maximum and stance phase minimum at all locations was significantly higher for steps with the three-ply sock than no sock (*p* = 0.000) (Figure 8).

## 4. Discussion

Socket fit sensing may enhance prosthetic care by providing quantitative feedback during patient training and by enhancing a practitioner’s capability to diagnose the source of socket fit problems. It may further the development of self-adjusting protheses to accommodate changing residual limb size and environmental conditions (e.g., different terrains) by serving as a feedback signal within closed-loop automatically adjusting sockets. The described method for fabrication of a magnetically permeable target and integration into an off-the-shelf prosthetic liner enhances the feasibility of using inductive sensing for these objectives.

The consistency in measurement across the 38 cm × 53 cm e-FPPC sheet was a result of the consistent thickness in the layers of ferrous polymer, clean polymer, and elastic fabric achieved from the careful fabrication procedure. During development, we found that the key variables to control in the fabrication process were the temperature of the iron/polymer mixture before pouring it into the mold; elimination of bubbles in the polymer before pouring; maintenance of a consistent pouring rate of the polymer as it is was spread into the mold; and strict control of the temperature and time heating the FPPC when forming the e-FPPC. Means for managing these challenges are presented in more detail in Appendix A. Quality of the sensor signal may depend on the skills of the person doing the fabrication. To reduce variability, equipment for automated fabrication is preferable. The detailed descriptions provided in Appendix A.1 help define the needs of such equipment.

The ferrous liner construct endured 28 days of repetitive wear in a shoe in part because the mechanical properties of the FPPC were well matched to the surrounding materials. In development of the SEEPS polymer in our prior efforts, we noted that the polymer stiffness could be tuned to the elastomer to which it is to be attached. The tensile elasticity of the ferrous polymer was 282 kPa, within the range of commercial elastomeric liners (124–309 kPa) [17,20]. This match minimized shear stress at their interface when subjected to an external load and allowed the very thin layer of highly elastic fabric between them to serve as a bonding platform to keep the layers together. No delamination problems were evident in the 28-day wear samples. Stresses experienced in the shoe under the heel of an able-bodied person (reported up to 51 kPa shear stress and 358 kPa pressure [22]) approximate those at the limb–socket interface of prosthesis users (up to 57 kPa shear stress and 342 kPa pressure at the sites of interest here [23]). Long-term testing on prosthesis users with a wide range of limb and socket dimensions are needed to further assess durability, and to evaluate fatigue and creep. The effects of liner thickness, stiffness, and strength on calibration results should also be evaluated.

The increasingly negative distance offset observed over the first approximately 12 days of durability testing (Figure 6b) was likely a result of structural changes within the e-FPPC construct. The elastic fabric on the socket side of the e-FPPC may have reduced its percent air volume by plastically deforming (compression) or integrating deeper into the clean polymer as a result of the repetitively applied stress during walking. However, since the elastic fabric and the clean polymer together were only 0.55 mm in thickness, it is unlikely that the approximately 0.50-mm distance offset measured (Figure 6b) was exclusively from this source. The ferrous polymer is slightly compressible (Poisson ratio of 0.4947 as deemed in prior testing [20]). The ferrous polymer layer may have compressed into a denser structure or undergone a chemical change, which may increase its wt %, increasing its signal intensity per unit distance and possibly contributing to a more negative distance offset. However, we did not see a meaningful distance offset in our prior work where we conducted in-shoe wear testing on just a fabric-ferrous polymer-fabric construct [20]. Independent of the source, the result indicates that the ferrous liner should be pre-conditioned before participant use, or that the participant should wear the liner for about two weeks before sensor data are collected. After the in-shoe tests were completed, we sectioned and imaged the samples under a microscope and compared them with sections from the original e-FPPC sheet, but the resolution using this method was insufficient to measure a change in thickness of the ferrous polymer layer. The hypothesized mechanical changes within the e-FPPC construct need to be tested with an alternative method to help identify sources of the distance offset. We further studied the images and did not identify any delamination or other degradation. We cut a cross-section of the liner and monitored using a video camera the motion between layers under cyclic shear and compression. No separation was apparent between layers.

It is not known why the distance offset during the durability test is so much different for the Day 24 test compared with the Day 21 or 28 test (Figure 6b). Because the result was observed in both test samples, we suspect that it was an error in the calibration device or execution of the test procedure. A more advanced calibration system, for example one that adjusted the distance to the sample using computer control rather than manual control, similar to a mechanical testing system, may reduce these errors. Such improvement may also provide a means for calibration of the sensor to applied pressure and thus a means to quantify interface stress.

Results from amputee participant testing demonstrated that adding a sock increased stance phase minima and swing phase maxima. The increase in sensed distance when the participant wore the three-ply sock (mean distance of 0.91 mm) was comparable to measurements of the thickness of three-ply socks reported previously under stance phase loading conditions [24] (distance range of 0.60 to 1.17 mm). The sock kept the liner a further distance from the socket wall. The difference between the swing phase maxima and stance phase minima at the posterior mid-limb locations was lower than other locations (Figure 8). We expect this result reflects the minimal loss of contact for the posterior soft tissue region. The sensor faithfully measures the distance between the socket wall and liner in a direction perpendicular to the socket surface, though we recognize that at some locations (e.g., other than posterior mid-limb) the region of the target monitored may change over the course of swing phase since the liner may line up in the socket differently. A uniform thickness e-FPPC ensures this is not problematic towards conversion of sensor data to distance data.

A next step is to calibrate the sensor for Condition (iii) in Figure 1b—presence of contact pressure between the target and socket. A controlled actuator that maintains the antenna parallel with the target during compression will be needed to execute this calibration on e-FPPC samples. The system shown in Figure 5 is inadequate because the lever arm bends upon contact with the sample. A material testing machine modified for this purpose may accomplish this objective. Potentially, a calibration curve relating sensed distance to applied pressure can be identified.

## 5. Conclusions

The developed fabrication methods achieved a consistent target thickness and depth within an elastic ferrous polymer/clean polymer construct. When the construct was attached to a prosthetic liner and worn in the shoe of an able-bodied participant, it demonstrated a consistently-shaped calibration curve over 28 days of wear but an inconsistent distance reference for the first 12 days of wear. Before being used in clinical application for liner-to-socket distance monitoring, new ferrous liners may need to be pre-worn so as to achieve a consistent distance reference. Although no damage or delamination was apparent in any of the samples, long-term testing on prosthesis users is needed to test durability for regular clinical use.

## Figures and Tables

**Table 1:** Substances used to make an embedded ferrous polymer/polymer construct for one liner.

Substance	Product	Manufacturer	Quantity per Liner
Mold release agent	Ease Release 200	Mann	10-s spray time (aerosol)
Polymer pellets	SEPTON™ 4044	Kuraray	10 g
Mineral oil	White Mineral Oil Light	McMaster-Carr	50 g
Iron powder	≥99% trace metals basis; 6–10 μm particle size	Sigma Aldrich	340 g
Elastic fabric	Light Weight Soft Lycra Spandex 4 Way Stretch Peach LY712	Discount Fabric	45.7 cm × 45.7 cm (×2)
Thread	100% Polyester Sewing Thread	Ilauke	150 cm
Silicone adhesive	Sil-Poxy	Smooth On	30 g

## Appendix A

### Appendix A.2. Attachment to the off-the-Shelf Liner

**Table A1:** Equipment and supplies used for fabrication.

Equipment (Quantity)	Requirements	Manufacturer	Model No.
Hot plates under mold base (2)	30.5 cm × 30.5 cm, ≥300 °C	Torrey Pines Scientific	HP51A
Hot plate for mixing	17.8 cm × 17.8 cm, ≥280 °C	Fisher Scientific	Sp88857200
Oven	20.3 cm × 20.3 cm × 30.5 cm, ≥250 °C	Precision Scientific	Model 19
Vacuum pump for oven	0.33 HP, ≥27 in Hg	Fillauer	227020
Sewing machine	Serger style for durable stretchy stitches	Brother	Lock 1034D
Socket fabrication jig	Provides mount for foam positive	Fillauer	LJ-100
